# Microbial Therapeutics in Oncology: A Comprehensive Review of Bacterial Role in Cancer Treatment

**DOI:** 10.7759/cureus.70920

**Published:** 2024-10-06

**Authors:** Radha Kunjalwar, Akshunna Keerti, Achal Chaudhari, Kaushik Sahoo, Supriya Meshram

**Affiliations:** 1 Microbiology, Jawaharlal Nehru Medical College, Datta Meghe Institute of Higher Education and Research, Wardha, IND; 2 Internal Medicine, Jawaharlal Nehru Medical College, Datta Meghe Institute of Higher Education and Research, Wardha, IND

**Keywords:** bacterial therapy, cancer treatment, genetic engineering, immune modulation, microbial oncology, tumor targeting

## Abstract

Conventional cancer therapies, including chemotherapy, radiotherapy, and immunotherapy, have significantly advanced cancer treatment. However, these modalities often face limitations such as systemic toxicity, lack of specificity, and the emergence of resistance. Recent advancements in genetic engineering and synthetic biology have rekindled interest in using bacteria as a novel therapeutic approach in oncology. This comprehensive review explores the potential of microbial therapeutics, particularly bacterial therapies, in the treatment of cancer. Bacterial therapies offer several unique advantages, such as the ability to selectively target and colonize hypoxic and necrotic regions of tumors, areas typically resistant to conventional treatments. The review delves into the mechanisms through which bacteria exert antitumor effects, including direct tumor cell lysis, modulation of the immune response, and delivery of therapeutic agents like cytotoxins and enzymes. Various bacterial species, such as *Salmonella*, *Clostridium*, *Lactobacillus*, and *Listeria*, have shown promise in preclinical and clinical studies, demonstrating diverse mechanisms of action and therapeutic potential. Moreover, the review discusses the challenges associated with bacterial therapies, such as safety concerns, immune evasion, and the need for precise targeting, and how recent advances in genetic engineering are being used to overcome these hurdles. Current clinical trials and combination strategies with conventional therapies are also highlighted to provide a comprehensive overview of the ongoing developments in this field. In conclusion, while bacterial therapeutics present a novel and promising avenue in cancer treatment, further research and clinical validation is required to fully realize their potential. This review aims to inspire further exploration into microbial oncology, paving the way for innovative and more effective cancer therapies.

## Introduction and background

Cancer remains one of the most significant global health challenges, with millions of new cases diagnosed annually. Despite considerable progress in oncology, conventional treatment modalities, chemotherapy, and radiotherapy, and surgery, continue to dominate clinical practice [1]. Chemotherapy, involving the systemic administration of cytotoxic drugs, targets rapidly dividing cancer cells but often lacks specificity. This non-selective action affects healthy proliferating cells, leading to a variety of adverse effects, including myelosuppression, gastrointestinal disturbances, and increased vulnerability to infections. The toxicity associated with chemotherapy, along with the potential for drug resistance, limits its long-term efficacy and reduces patient compliance [2]. Radiotherapy, another cornerstone of cancer treatment, employs high-energy radiation to damage the DNA of cancer cells, halting their proliferation and inducing cell death. While effective for localized tumors, radiotherapy’s therapeutic window is constrained by the risk of damaging adjacent healthy tissues. This collateral damage can result in significant complications such as fibrosis, pneumonitis, and, in some instances, secondary cancers. These risks necessitate the use of precise targeting techniques to minimize harm to healthy tissues [3].

Immunotherapy has revolutionized cancer treatment by harnessing the body's immune system to identify and destroy tumor cells. Therapies like immune checkpoint inhibitors, adoptive cell transfer, and cancer vaccines have shown remarkable success in treating malignancies such as melanoma, non-small cell lung cancer, and certain lymphomas [4]. However, immunotherapy is not without its challenges, including immune-related adverse events, variability in patient responses, and the development of resistance mechanisms. Despite these hurdles, immunotherapy represents a promising shift in cancer care, though its limitations underscore the need for more innovative, targeted therapies [5]. The concept of using microbes in medicine has a rich history, with the discovery of antibiotics transforming the treatment of infectious diseases. Yet, the potential of microbes, particularly bacteria, in cancer therapy has only recently regained attention [6]. Bacterial therapy in oncology dates back to the late 19th century, when Dr. William Coley, often regarded as the "father of cancer immunotherapy," observed tumor regression in cancer patients following injections of live Streptococcus pyogenes. Though this early approach showed some success, it was largely abandoned with the advent of standardized treatments such as chemotherapy and radiotherapy [6].

In recent years, advancements in genetic engineering and synthetic biology have reignited interest in the use of bacteria as therapeutic tools against cancer. Modern bacterial therapies involve genetically modified bacteria that selectively target and proliferate within tumor tissues [7]. These bacteria exert antitumor effects through various mechanisms, including direct oncolysis, modulation of the tumor microenvironment, and delivery of therapeutic agents like cytotoxins, enzymes, or immunomodulators. Unlike traditional therapies, bacterial therapeutics have the unique ability to penetrate and thrive in the hypoxic and necrotic regions of tumors-areas typically resistant to both chemotherapy and radiotherapy [7]. This resurgence in bacterial therapy research is driven by the urgent need to overcome the limitations of existing cancer treatments. Bacteria's inherent characteristics, such as their capacity to colonize specific tissues and their amenability to genetic manipulation, position them as promising candidates for novel cancer therapies. The emerging field of microbial oncology seeks to harness these properties, potentially offering more effective, targeted, and personalized treatment options for cancer patients [8].

This review aims to provide a comprehensive analysis of the current state and future prospects of bacterial therapeutics in cancer treatment. We will examine the diverse mechanisms through which bacteria exert antitumor effects, including direct tumor cell killing, immune system modulation, and the delivery of therapeutic agents. Furthermore, we will highlight various bacterial species that have shown promise in preclinical and clinical studies, exploring their unique characteristics, mechanisms of action, and therapeutic potential. Additionally, this review will address strategies for enhancing the safety and efficacy of bacterial therapies, including genetic modifications, combinations with existing cancer treatments, and innovative delivery systems.

## Review

Mechanisms of bacterial anticancer activity

Bacterial therapies in oncology employ a variety of mechanisms to exert anticancer effects, which can be categorized into direct antitumor action, immune modulation, enzyme-mediated prodrug therapy, and gene therapy using bacterial vectors [9]. One of the most advantageous aspects of bacteria in cancer therapy is their ability to selectively target and colonize tumors, owing to their unique biological properties. Certain bacterial strains, such as *Clostridium* and *Salmonella*, preferentially thrive in the hypoxic and nutrient-rich environments characteristic of tumors, enabling effective colonization. Research has demonstrated that engineered *E. coli* strains can localize significantly within tumor tissues instead of normal tissues, enhancing their therapeutic potential [10]. The direct antitumor effects of bacteria operate through multiple mechanisms. One key mechanism is nutrient competition, where bacteria outcompete tumor cells for vital nutrients, inhibiting tumor growth. Additionally, some bacterial strains secrete cytotoxic compounds or toxins that directly induce cancer cell death. For instance, *Clostridium* species release proteins that trigger apoptosis in tumor cells, contributing to their anticancer activity [11]. Bacteria also play a crucial role in modulating the immune response against tumors. They can stimulate the immune system to recognize and destroy cancer cells by activating various immune pathways. This activation leads to the recruitment of immune cells to the tumor site and enhances antigen presentation, which is vital for a robust immune response [12]. Moreover, bacterial colonization of tumors can alter the tumor microenvironment, making it more susceptible to immune attacks. By inducing inflammation and modifying cytokine profiles, bacteria can improve the efficacy of other treatments, such as immune checkpoint inhibitors and cancer vaccines. This immune modulation is promising for improving cancer treatment outcomes [13]. An innovative approach known as bacterial-directed enzyme prodrug therapy (BDEPT) harnesses bacteria to convert non-toxic prodrugs into active cytotoxic agents, specifically within tumor sites. This two-step process minimizes systemic toxicity while maximizing local drug activation. For example, engineered *E. coli* strains have been shown to convert prodrugs like glycyrrhizic acid into their active forms, significantly reducing tumor growth in preclinical models [14]. One notable example is the nitroreductase (NTR) enzyme, derived from *Clostridium* species, which converts prodrugs such as CB1954 into highly toxic derivatives that induce cell death in proliferating and non-proliferating cancer cells. This specificity enhances treatment efficacy and reduces the adverse effects associated with conventional chemotherapy [15]. Bacteria can also serve as vectors for gene therapy, where they deliver therapeutic genes directly to tumors, providing a targeted cancer treatment approach. This method enhances localized treatment effects while minimizing off-target impacts on healthy tissues. Experimental models have shown that genetically modified bacteria, engineered to express therapeutic proteins or enzymes, can lead to significant tumor regression [16]. For instance, studies involving *Salmonella* strains engineered to express cytotoxic agents have yielded promising results in preclinical cancer models. Tum-specific promoters ensure that these therapeutic genes are activated primarily within cancerous tissues, enhancing both safety and efficacy. Overall, applying genetically engineered bacteria for gene therapy represents a cutting-edge strategy in the fight against cancer [17].

Bacterial species in cancer therapy

Bacterial species are emerging as promising candidates for cancer therapy due to their unique properties, enabling them to target and destroy tumor cells selectively. This section examines several vital bacterial species, focusing on their mechanisms of action, therapeutic potential, and clinical applications [18]. *Salmonella typhimurium* has garnered significant attention for its potential in cancer treatment, particularly its ability to be genetically modified for enhanced safety and efficacy. These modifications allow the bacteria to selectively proliferate within tumors, leading to tumor cell death through direct lysis and the induction of an immune response [19]. Additionally, *Salmonella* can serve as a vector for delivering therapeutic agents directly to tumor sites, thereby enhancing the overall efficacy of cancer treatments. Preclinical studies have demonstrated that *Salmonella* targets various tumor types in animal models. Early-phase clinical trials have also shown promise, with some patients experiencing tumor regression following treatment with engineered *Salmonella* strains. However, challenges remain regarding optimizing dosing and delivery methods to maximize therapeutic effects while minimizing side effects [19]. *Clostridium* species, particularly *Clostridium* novyi-NT, are well-known for their ability to thrive in hypoxic environments typical of many solid tumors. These anaerobic bacteria selectively colonize necrotic tumor tissues, germinating and producing toxins that lead to tumor cell lysis. This targeting mechanism is especially advantageous for treating tumors resistant to conventional therapies [20]. *Clostridium* novyi-NT has shown significant promise in preclinical studies, leading to its evaluation in clinical trials for treatment-resistant tumors. However, challenges include ensuring adequate bacterial colonization within tumors and managing potential side effects associated with bacterial therapies. Researchers are exploring combining *Clostridium* therapy with conventional treatments like chemotherapy to enhance efficacy [20]. *Lactobacillus* species are recognized for their immunomodulatory effects, which can bolster the body's immune response against tumors. These probiotics play a crucial role in maintaining gut microbiota balance during cancer treatments, potentially reducing the side effects of chemotherapy and radiotherapy. Probiotic supplementation is currently being investigated as an adjuvant therapy in cancer treatment protocols. By enhancing immune function and reducing inflammation, probiotics may improve patient outcomes when used alongside traditional cancer therapies [21]. *Listeria* monocytogenes is a bacterium noted for its ability to induce potent immune responses against tumors. It infects tumor cells and presents tumor antigens to the immune system, enhancing the body's capacity to recognize and attack cancer cells. This mechanism has been leveraged in developing *Listeria*-based vaccines to treat various cancers. Clinical trials involving *Listeria*-based vaccines have yielded promising results in eliciting robust antitumor immune responses. These vaccines are designed to be combined with other immunotherapy or chemotherapy to improve treatment efficacy [22]. The potential applications of these bacterial species in cancer therapy are summarized in Table 1.

**Table 1:** Bacterial species in cancer therapy

Bacterial Species	Type	Mechanism of Action	Cancer Types Targeted	Clinical Status
Clostridium novyi-NT [23]	Anaerobic	Spore-forming bacteria that germinate in hypoxic tumors	Glioma, Breast Cancer, Colorectal Cancer	Phase I Clinical Trials
Salmonella typhimurium [24]	Facultative Anaerobic	Tumor-targeting via preferential growth in tumors induces an immune response	Melanoma, Breast Cancer, Colon Cancer	Phase I/II Clinical Trials
Bifidobacterium breve [25]	Anaerobic	Selective colonization of tumors, modulation of the immune system	Solid Tumors	Preclinical Studies
Escherichia coli Nissle 1917 [26]	Facultative Anaerobic	Secretion of therapeutic proteins, drug delivery	Colorectal Cancer	Preclinical Studies
Listeria monocytogenes [27]	Facultative Anaerobic	Infection and induction of antitumor immunity	Pancreatic Cancer, Melanoma, Cervical Cancer	Phase I/II Clinical Trials
Bacillus Calmette-Guérin (BCG)	Aerobic	Induces immune response in bladder urothelium	Bladder Cancer	FDA-Approved for Bladder Cancer Therapy
Clostridium sporogenes [28]	Anaerobic	Enzyme-prodrug therapy, the conversion of prodrugs into active drugs within the tumor	Solid Tumors	Preclinical Studies
Mycobacterium bovis [29]	Aerobic	Activation of immune response, non-specific immunostimulation	Bladder Cancer	FDA-Approved for Bladder Cancer Therapy
Streptococcus pyogenes [30]	Facultative Anaerobic	Oncolytic bacterial therapy, induction of inflammation, and immune response	Melanoma, Breast Cancer	Preclinical Studies
Lactobacillus casei [31]	Facultative Anaerobic	Immunomodulation and enhancement of host immunity	Colorectal Cancer	Preclinical Studies

Clinical applications and trials

Numerous clinical trials are currently investigating the use of bacterial therapies in oncology, focusing on their ability to target tumors and enhance treatment efficacy selectively. These trials explore various bacterial strains, including *Salmonella typhimurium*, *Clostridium spp.*, and genetically engineered microbes [32]. For instance, the *Salmonella typhimurium* strain VNP20009 is being evaluated for its tumor-targeting properties and potential to enhance immune responses against cancer. Early results indicate promising tumor regression and survival benefits in patients with solid tumors. Additionally, genetically modified strains like Lactococcus lactis are under investigation as vehicles for delivering therapeutic agents directly to tumors, demonstrating safety and preliminary efficacy in early-phase trials [32]. Initial outcomes from these trials show that bacterial therapies can lead to significant tumor regression and improved patient survival rates. The future of bacterial therapies in oncology appears promising, particularly as advancements in genetic engineering allow for more targeted and effective treatments. As researchers continue exploring the mechanisms by which bacteria can influence tumor biology, we can expect many innovative therapeutic strategies to emerge [33]. Several case studies illustrate bacterial therapies' successes and challenges in clinical settings. One notable success involved the intentional introduction of Streptococcus pyogenes to a cancer patient, resulting in rapid tumor regression. This case highlights the potential of using live bacteria as immunotherapeutic agents to stimulate an immune response against tumors. Another example is the use of Bacillus Calmette-Guérin (BCG) therapy for bladder cancer, which has demonstrated effectiveness in reducing recurrence rates among patients, establishing a precedent for bacterial therapies in oncology [8,34]. However, not all cases have been successful; some trials have faced challenges related to safety, including severe infections or adverse immune responses. These setbacks emphasize the need for careful patient selection and monitoring during treatment. The lessons learned from these translational research efforts underscore the importance of understanding the tumor microenvironment and optimizing bacterial strains for enhanced targeting and reduced toxicity. Ongoing studies aim to refine these approaches further to improve clinical outcomes [35]. Despite the potential of bacterial therapies, several challenges must be addressed before widespread clinical adoption can occur. Safety concerns regarding human use of live bacteria are paramount, particularly concerning the risk of infections. Regulatory frameworks for approving such therapies are still evolving, complicating their clinical implementation and necessitating thorough evaluation processes to ensure patient safety [36]. Large-scale production and standardization challenges pose significant hurdles for developing bacterial therapeutics. Producing these therapies at scale while ensuring consistent quality and potency is critical for meeting regulatory requirements [37]. Standardizing manufacturing processes is essential for compliance and ensuring patients receive safe and effective treatments. In summary, while microbial therapeutics show great promise in oncology, ongoing clinical trials and case studies and addressing inherent challenges will be vital for their successful integration into standard cancer treatment protocols [37]. Clinical applications and trials of bacterial therapeutics in cancer treatment are shown in Table 2.

**Table 2:** Clinical applications and trials of bacterial therapeutics in cancer treatment

Bacterial Species/Strain	Cancer Type	Therapeutic Approach	Mechanism of Action	Clinical Trial Phase
Clostridium novyi-NT [38]	Glioblastoma	Intratumoral Injection	Tumor lysis in hypoxic environments, induction of immune response	Phase I
Salmonella typhimurium VNP20009 [39]	Melanoma, Renal Cancer	Intravenous Injection	Selective accumulation in tumors, induction of inflammation	Phase I
Listeria monocytogenes (ADXS11-001) [40]	Cervical Cancer	Live-attenuated strain secreting HPV E7 protein	Activation of T-cell-mediated immune response	Phase II
Bacillus Calmette-Guérin (BCG) [41]	Bladder Cancer	Intravesical Instillation	Non-specific immune activation, induction of local immune response	FDA-Approved
Escherichia coli Nissle 1917 [42]	Colorectal Cancer	Oral Administration of Genetically Modified Strain	Delivery of therapeutic proteins, modulation of gut microbiota	Preclinical
Streptococcus pyogenes (OK-432) [43]	Head and Neck Cancer	Intratumoral Injection	Induction of tumor-specific immune response	Phase I/II
Listeria monocytogenes (CRS-207) [44]	Pancreatic Cancer	The live-attenuated strain expressing mesothelin	Mesothelin-specific immune activation	Phase II
Clostridium sporogenes [28]	Solid Tumors	Spore-forming bacteria combined with enzyme-prodrug therapy	Localized conversion of prodrug to active drug within the tumor	Preclinical
Bifidobacterium longum [45]	Colon Cancer	Oral Administration of Genetically Modified Strain	Colonization and secretion of therapeutic agents within the tumor	Phase I
Mycobacterium bovis (BCG) [46]	Non-muscle Invasive Bladder Cancer	Intravesical Instillation	Activation of local immune response	FDA-Approved
Salmonella typhimurium A1-R [47]	Sarcoma	Intravenous Injection	Preferential growth in tumors, reduction of metastasis	Preclinical
Clostridium butyricum MIYAIRI 588 [48]	Colorectal Cancer	Oral Administration	Modulation of gut microbiota, enhancement of host immunity	Phase I
Clostridium difficile (C. diff. TcdA- TcdB-) [49]	Pancreatic Cancer	Intravenous Injection	Oncolytic effect by inducing apoptosis in cancer cells	Preclinical
Salmonella typhimurium (VNP20009) [50]	Melanoma, Renal Cancer	Intravenous Injection	Tumor colonization, tumor lysis	Phase I
Lactobacillus rhamnosus GG [51]	Gastrointestinal Cancers	Oral Administration	Modulation of gut microbiota, enhancement of immune response	Phase II

Combination strategies

Integrating bacterial therapies with traditional cancer treatments has shown significant potential in enhancing therapeutic efficacy. This section explores the synergistic effects of combining bacteria with chemotherapy, immunotherapy, and nanotechnology [52]. Combining bacterial therapy with chemotherapy and radiotherapy can produce synergistic effects that enhance tumor destruction. Bacteria can selectively target and invade tumors, leading to localized inflammation that may increase the effectiveness of conventional therapies [8]. For instance, studies have demonstrated that *Salmonella typhimurium* can enhance the cytotoxic effects of doxorubicin in tumor models, resulting in improved survival rates in preclinical settings. *Clostridium* novyi-NT, an anaerobic bacterium, has shown promise when combined with chemotherapy, as it can induce necrosis within tumors, making them more susceptible to drug penetration [8]. Several preclinical studies have illustrated the benefits of these combination strategies. For example, a study involving *Clostridium* species showed that their presence could significantly reduce tumor size when used alongside standard chemotherapeutic agents. Furthermore, clinical trials explore these combinations, with early results indicating improved outcomes compared to monotherapy approaches [53]. Combining bacterial therapies with immune checkpoint inhibitors (ICIs) is a burgeoning area of research. ICIs, such as those targeting PD-1 and CTLA-4, enhance the immune system's ability to recognize and attack cancer cells. Bacteria can modulate the immune response by altering gut microbiota composition, which is linked to improved responses to ICIs. For instance, studies suggest that specific gut microbial signatures can predict patient responses to ICIs, indicating a potential role for bacteria in enhancing immunotherapeutic efficacy [54]. Moreover, bacterial therapies may help overcome immune resistance observed in some tumors. By inducing a local immune response through bacterial infection or delivering immune-stimulating agents directly to the tumor site, these therapies can potentially reactivate T-cell responses that the tumor microenvironment has suppressed. Research indicates that certain probiotics may enhance the effectiveness of ICIs by promoting T-cell activation and improving overall survival rates in patients undergoing immunotherapy [55]. Bacteria can also be effective carriers for targeted nanoparticle delivery systems in cancer therapy. This innovative approach leverages the natural homing abilities of bacteria to deliver therapeutic nanoparticles directly to tumor sites while minimizing systemic toxicity. For example, engineered bacteria can be loaded with nanoparticles that release chemotherapeutic or imaging agents upon reaching the tumor microenvironment [56]. Recent advancements in nanotechnology have led to the development of multifunctional nanoparticles that can be combined with bacterial vectors. These nanoparticles can be designed to respond to specific stimuli within the tumor microenvironment, such as pH changes or enzyme activity, ensuring precise drug release at the desired location. This strategy enhances therapeutic efficacy and reduces side effects associated with conventional systemic treatments [57]. Combination strategies of bacterial therapeutics with conventional cancer treatments are shown in Table 3.

**Table 3:** Combination strategies of bacterial therapeutics with conventional cancer treatments

Bacterial Species/Strain	Combination Partner	Cancer Type	Mechanism of Action	Clinical Trial Phase
Salmonella typhimurium VNP20009 [58]	Chemotherapy (Cisplatin)	Melanoma, Colorectal Cancer	Enhanced tumor lysis, increased tumor-specific immune response	Preclinical
Clostridium novyi-NT [59]	Immune Checkpoint Inhibitors (anti-PD-1)	Glioblastoma, Breast Cancer	Tumor lysis, potentiation of immune checkpoint blockade	Phase I
Listeria monocytogenes (ADXS31-164) [60]	Radiation Therapy	Head and Neck Cancer	Radiation-induced immunogenic cell death, enhanced immune response	Phase II
Bacillus Calmette-Guérin (BCG) [61]	Immunotherapy (IFN-α)	Bladder Cancer	Enhanced immune response, increased cytokine production	Phase II
Escherichia coli (strain Nissle 1917) [62]	Probiotics + Chemotherapy	Colorectal Cancer	Modulation of gut microbiota, improved chemotherapy efficacy	Preclinical
Listeria monocytogenes (CRS-207) [44]	Chemotherapy (Gemcitabine)	Pancreatic Cancer	Enhanced antigen presentation, increased cytotoxic T-cell response	Phase II
Clostridium butyricum MIYAIRI 588 [63]	Immunotherapy (anti-CTLA-4)	Colorectal Cancer	Modulation of gut microbiota, enhanced checkpoint blockade efficacy	Preclinical
Salmonella typhimurium (A1-R) [64]	Radiation Therapy	Osteosarcoma	Synergistic tumor lysis, inhibition of metastasis	Preclinical
Listeria monocytogenes (LM-NP2) [27]	Chemotherapy (Paclitaxel)	Breast Cancer	Improved drug delivery, increased apoptosis in cancer cells	Preclinical
Mycobacterium bovis (BCG) [65]	Chemotherapy (Mitomycin C)	Bladder Cancer	Increased cytotoxicity, augmented immune response	Phase III
Salmonella typhimurium (VNP20009) [66]	Targeted Therapy (Anti-EGFR)	Colorectal Cancer	Tumor-specific colonization, inhibition of EGFR signaling	Preclinical
Streptococcus pyogenes (OK-432) [43]	Chemotherapy (Cisplatin, 5-FU)	Head and Neck Cancer	Enhanced immune response, reduced tumor size	Phase II
Clostridium difficile (TcdA- TcdB-) [67]	Immune Checkpoint Inhibitors (anti-CTLA-4, anti-PD-1)	Pancreatic Cancer	Oncolytic activity, potentiation of immune checkpoint blockade	Preclinical
Lactobacillus casei Shirota [68]	Chemotherapy (Capecitabine)	Colorectal Cancer	Modulation of gut microbiota, improved chemotherapy efficacy	Phase II
Clostridium sporogenes [69]	Prodrug (5-Fluorocytosine)	Solid Tumors	Tumor-localized drug activation, reduced systemic toxicity	Preclinical

Future directions and innovations

As microbial therapeutics in oncology evolve, several innovative approaches are emerging to enhance the efficacy and personalization of bacterial therapies. This section explores advances in genetic engineering, personalized bacterial therapy, and the integration of artificial intelligence in optimizing bacterial treatments for cancer [10]. Recent advancements in genetic engineering, particularly through CRISPR technology, have revolutionized the modification of bacterial genomes. CRISPR systems can be tailored for various bacterial species, enhancing their therapeutic potential in cancer treatment. Techniques such as CRISPR nickases and base editing offer alternatives that may reduce off-target effects and improve editing efficiency in bacteria [70]. Additionally, synthetic biology tools are employed to design bacteria capable of producing therapeutic agents or responding to specific stimuli within the tumor microenvironment. This optimization allows for a more targeted approach to cancer therapy, increasing the likelihood of successful treatment outcomes [70]. Emerging technologies are also being developed to improve the ability of bacteria to target tumors. Engineered bacteria can be designed to respond to tumor-specific signals or environmental conditions, such as hypoxia, allowing for localized delivery of therapeutic agents. Furthermore, advancements in biosensors and bioengineering enable real-time monitoring of bacterial behavior within tumors, enhancing treatment precision and efficacy [10]. Personalized medicine is becoming increasingly relevant in cancer therapy, and bacterial treatments are no exception. By analyzing individual patient microbiomes and tumor characteristics, researchers can select or engineer specific bacterial strains more likely to elicit effective immune responses or deliver therapeutic payloads tailored to the patient's unique tumor profile. This personalized approach aims to enhance treatment efficacy while minimizing adverse effects, making it a promising avenue for improving patient outcomes [71]. Moreover, the human microbiome plays a critical role in shaping immune responses and influencing the effectiveness of cancer therapies. Understanding how different microbiome compositions affect bacterial therapy outcomes can lead to more effective treatment strategies. For instance, certain gut microbiota have been shown to enhance the efficacy of immunotherapies, suggesting that integrating microbiome analysis into treatment planning could optimize bacterial therapies by aligning them with the patient's unique microbial landscape [72]. Artificial intelligence (AI) is increasingly used to predict patient responses to bacterial therapies. Machine learning algorithms can analyze vast datasets from clinical trials and patient records to identify patterns correlating with successful treatment outcomes. This predictive capability allows for better patient stratification and personalized treatment plans, ensuring patients receive the most appropriate therapy based on their circumstances [73]. Additionally, AI-driven models are being developed to assist in selecting the most suitable bacterial strains for individual patients based on their unique tumor characteristics and microbiome profiles. These models can integrate genomic data, treatment history, and other relevant factors to recommend optimal therapeutic strategies. By harnessing the power of AI, researchers aim to enhance the precision of microbial oncology treatments, ultimately leading to improved patient care [74]. Future directions and innovations in bacterial therapeutics for cancer treatment are shown in Table 4.

**Table 4:** Future directions and innovations in bacterial therapeutics for cancer treatment

Innovation/Strategy	Description	Potential Advantages	Current Status	Future Research Focus
Genetically Engineered Bacteria [75]	Bacteria modified to express therapeutic proteins, cytokines, or oncolytic factors	Targeted delivery, reduced off-target effects	Preclinical	Optimization of genetic constructs, safety assessment
Bacterial-Derived Nanoparticles [76]	Utilization of bacterial outer membrane vesicles (OMVs) or bacterial-based nanoparticles for drug delivery	Enhanced drug stability, targeted delivery, immune modulation	Preclinical	Scalability, targeting specificity, biocompatibility
Bacteria-Mediated Immune Modulation [77]	Use of bacteria to modulate the tumor microenvironment and enhance immune checkpoint inhibitor efficacy	Improved response to immunotherapy, reduced immune evasion	Early Clinical Trials	Identification of optimal bacterial strains
Synthetic Biology Approaches [78]	Development of synthetic circuits in bacteria for controlled release of therapeutics	Precise control over therapeutic action, programmable responses	Preclinical	Circuit optimization, regulatory challenges
Bioengineered Probiotics [79]	Probiotics engineered to deliver anti-cancer agents or modulate the gut microbiota to enhance treatment response	Non-invasive administration, improved gut health	Preclinical	Clinical translation, long-term safety studies
Bacterial Spore-Based Therapies [80]	Use of bacterial spores that germinate specifically within hypoxic tumor environments	Selective activation in tumors, reduced systemic toxicity	Preclinical	Optimization of spore-forming species, safety studies
Oncolytic Bacteria Therapy [81]	Bacteria designed to directly lyse tumor cells and induce an immune response	Direct tumor killing, induction of systemic anti-tumor immunity	Early Clinical Trials	Enhancing oncolytic potency, minimizing side effects
Bacterial Gene Therapy Vectors [82]	Bacteria as vectors to deliver gene-editing tools (e.g., CRISPR/Cas9) into tumors	Precise genetic modifications within the tumor microenvironment	Research Phase	Safety, specificity, and delivery efficacy
Combined Modality Approaches [83]	Integration of bacterial therapies with chemotherapy, radiotherapy, or targeted therapies	Synergistic effects, overcoming resistance to single modalities	Early Clinical Trials	Optimization of combination protocols, clinical trials
Bacterial Toxins and Metabolites [84]	Use of bacterial toxins or metabolites with anticancer properties	Novel mechanisms of action, potential for new drug development	Preclinical	Identification of potent candidates, delivery methods
Microbiome-Based Predictive Biomarkers [84]	Utilization of patient microbiome profiles to predict response to bacterial therapies	Personalized therapy, improved patient outcomes	Research Phase	Validation in large cohorts, integration in clinical practice
Bacterial Therapy with CAR-T Cells [84]	Use of bacterial therapies to modulate the tumor microenvironment and enhance CAR-T cell efficacy	Enhanced T-cell infiltration, reduction of immune suppression	Research Phase	Optimization of bacterial strains, combination strategies
Development of Bacteria for Radiation Sensitization [84]	Engineering bacteria to enhance the effectiveness of radiation therapy	Lower radiation doses, reduced side effects	Preclinical	Identification of effective strains, clinical testing
Advanced Drug Delivery Systems [84]	Bacteria loaded with nanoparticles or therapeutic agents for localized delivery	Enhanced drug concentration in tumors, reduced systemic toxicity	Preclinical	Design of delivery systems, clinical translation
Synthetic Bacteria and Microbiota Engineering [84]	Creation of synthetic microbial communities tailored to individual patient needs	Customized therapy, reduction of adverse effects	Research Phase	Regulatory challenges, safety assessments

## Conclusions

Exploring microbial therapeutics in oncology represents a burgeoning field with significant potential to revolutionize cancer treatment. While conventional therapies such as chemotherapy, radiotherapy, and immunotherapy have significantly improved patient outcomes, their specificity, toxicity, and resistance limitations necessitate the search for more innovative approaches. Bacterial therapies offer a promising alternative with their unique ability to target and proliferate within the tumor microenvironment. The diverse mechanisms through which bacteria can exert anticancer effects-including direct oncolysis, modulation of immune responses, and delivery of therapeutic agents, highlight their versatility and potential as a complementary or standalone treatment modality. Despite these promising prospects, several challenges remain, including the need for enhanced safety, efficacy, and precision in targeting. Advances in genetic engineering, synthetic biology, and personalized medicine promise to address these challenges, paving the way for the successful integration of bacterial therapies into mainstream oncology. As research in this field progresses, rigorous clinical trials and regulatory frameworks will be essential to translate these innovative therapies from the laboratory to the clinic. Ultimately, microbial therapeutics may expand the arsenal of tools available to oncologists and offer new hope for patients facing difficult-to-treat cancers.
